# Eficácia e Segurança do Isolamento Adjuvante da Parede Posterior em Pacientes com Fibrilação Atrial Persistente: Uma Revisão Sistemática e Metanálise

**DOI:** 10.36660/abc.20240472

**Published:** 2025-01-22

**Authors:** João Vitor Levindo Coelho Novaes, David de Pádua Brasil, Flavia Maria de Freitas Faria, Isadora Soares Bicalho Garcia, Camila Ribeiro Pimenta, Nathalia Sernizon Guimarães, Marcus Vinicius Bolivar Malachias

**Affiliations:** 1 Faculdade Ciências Médicas de Minas Gerais Fundação Educacional Lucas Machado Belo Horizonte MG Brasil Faculdade Ciências Médicas de Minas Gerais - Fundação Educacional Lucas Machado, Belo Horizonte, MG – Brasil; 2 Faculdade de Ciências da Saúde Universidade Federal de Lavras Lavras MG Brasil Faculdade de Ciências da Saúde - Universidade Federal de Lavras, Lavras, MG – Brasil; 3 Departamento de Nutrição Universidade Federal de Minas Gerais Belo Horizonte MG Brasil Departamento de Nutrição - Universidade Federal de Minas Gerais, Belo Horizonte, MG – Brasil

**Keywords:** Ablação por Cateter, Fibrilação Atrial, Eletrofisiologia Cardíaca

## Abstract

**Fundamento:**

Em pacientes com fibrilação atrial (FA) persistente, a realização do isolamento da parede posterior (IPP) além do isolamento das veias pulmonares (IVP) é controversa.

**Objetivo:**

Comparar IVP mais IPP versus IVP exclusivo em pacientes com FA persistente.

**Métodos:**

Trata-se de uma revisão sistemática conduzida nas bases de dados PubMed (MEDLINE), Embase, LILACS, CENTRAL (Cochrane Library), e Clinicaltrials.gov por ensaios clínicos randomizados comparando IVP + IPP e IVP exclusivo e FA persistente. Os desfechos foram (i) recorrência de FA; (ii) recorrência de arritmias atriais, isto é, FA, taquicardia atrial, ou flutter atrial); (iii) complicações clínicas importantes (isto é, derrame ou tamponamento pericárdico; disfunção do nó sinusal ou fístula atrioesofágica); (iv) tempo médio de ablação. O risco de viés e a qualidade da evidência foram avaliados usando a ferramenta Cochrane de avaliação de risco de viés (RoB 2.0) e o GRADE, respectivamente. A significância estatística foi estabelecida em 5%, e análises por subgrupos e de sensibilidade foram realizadas.

**Resultados:**

Foram incluídos oito estudos e 1119 pacientes, dos quais 561 se submeteram a IVP+IPP. Durante o seguimento (12-24 meses), a recorrência de FA foi significativamente diminuída com IPP adjuvante (RR 0,66; IC 95%; 0,44-0,98). O composto de arritmias atriais recorrentes não difere significativamente (RR 0,83, IC 95% 0,65- 1,06). As complicações clínicas maiores (RR 0,81, IC95% 0,42-1,58) foram similares, e o IVP exclusivo foi associado a um tempo médio mais curto de procedimento (diferença média -23,37 minutos, IC 95% -30,23, -16,50).

**Conclusão:**

O IPP adjuvante parece efetivo em melhorar FA recorrente, mas não a recorrência de todas as arritmias atriais. O tempo de procedimento foi mais longo com IVP + IPP sem mudança significativa na segurança global. Mais estudos são necessários para investigar os benefícios em longo prazo.

## Graphical abstract

**Figura Central:**
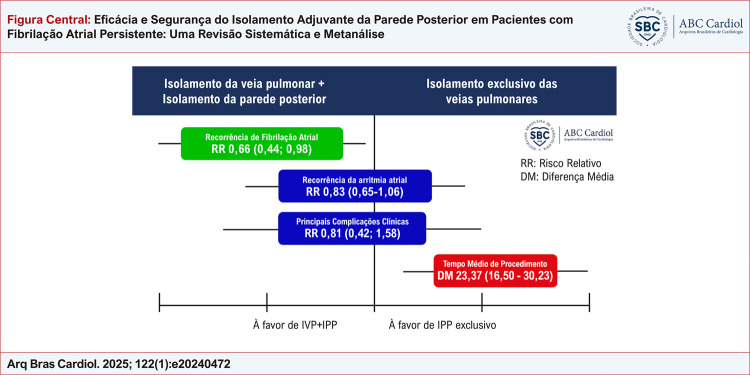
Eficácia e Segurança do Isolamento Adjuvante da Parede Posterior em Pacientes com Fibrilação Atrial Persistente: Uma Revisão Sistemática e Metanálise

## Introdução

A prevalência de Fibrilação Atrial (FA) está aumentando na população idosa, o que tem relação com síndrome metabólica. Tal aumento nas taxas de FA está associado com riscos consideráveis de mortalidade e morbidade, incluindo insuficiência cardíaca e acidente vascular cerebral.^1 - 3^ A ablação por cateter envolvendo o Isolamento das Veias Pulmonares (IVP) tende a ser menos efetivo em pacientes com FA persistente em comparação a pacientes com FA paroxística, como destacado em estudos anteriores.^4 , 5^

O início precoce do controle do ritmo está associado com progressão mais lenta da FA, e redução no risco de mortalidade global e cardiovascular em comparação ao controle da frequência cardíaca. De fato, técnicas de ablação se sobrepõem à terapia medicamentosa na manutenção do ritmo sinusal. No entanto, em estágios avançados da FA, mudanças e remodelamento no substrato atrial desloca mecanismos primários das veias pulmonares para outras estruturas dentro do átrio esquerdo.^6 , 7^

Acredita-se que a parede posterior do átrio esquerdo exerça um papel importante na fisiopatologia da FA persistente, dada a inclusão do feixe septo-pulmonar e sua conexão embriológica nas veias pulmonares.^8^ Apesar dos procedimentos de IVP, circuitos re-entrantes não relacionados às veias pulmonares podem persistir dentro do átrio esquerdo, diminuindo, assim, a eficácia do procedimento de ablação.^3 , 4 , 7 , 9^

Diante dessas considerações, o isolamento da parede posterior (IPP) ganhou força como uma técnica para o manejo da FA persistente. Após a publicação de metanálises^10 , 11^ comparando IPP adjuvante ao IVP em pacientes com FA persistente, ensaios clínicos randomizados (ECRs) subsequentes também foram relatados.^12 - 14^ Uma metanálise^15^ recente incluindo ensaios controlados randomizados e não randomizados, mostrou que o IPP pode melhorar significativamente a arritmia atrial e aumentar o tempo sem FA.

As diretrizes da Sociedade Europeia de Cardiologia de 2020 reconheceu o potencial da ablação extensiva, incluindo IPP, apesar de que a confirmação de sua eficácia continua pendente.^16^ Além disso, as diretrizes do ACC/AHA de 2023 considera incerta a avaliação de desfechos além do IVP.^17^Dada a importância clínica, propusemos realizar uma metanálise atualizada, considerando exclusivamente ECRs e usando a estrutura GRADE ( *Grading of Recommendations Assessment, Development and Evaluation* ) para avaliação da qualidade da evidência. Este estudo tem como objetivo avaliar a ablação por cateter envolvendo o IVP com IPP adjuvante versus IVP isolado em pacientes com FA persistente.

## Materiais e Métodos

Esta revisão sistemática e metanálise seguiu as recomendações das diretrizes Cochrane para revisões sistemáticas de intervenções e foi desenvolvida de acordo com o PRISMA.^18 , 19^ O protocolo da revisão foi registrado no Open Science Framework (doi.org/10.17605/OSF.IO/AZ5GU).

### Estratégia de busca

Para identificar ensaios clínicos que avaliaram a efetividade e a segurança da ablação por cateter envolvendo IVP com IPP adjuvante, em comparação ao IVP isolado em pacientes com FA persistente, a pesquisa foi conduzida em quatro bases de dados independentes: PubMed (MEDLINE), Embase, LILACS, Cochrane Central Register of Controlled Trials (CENTRAL) (Cochrane Library). Além disso, a estratégia de busca incluiu ensaios clínicos registrados no Clinicaltrials.gov junto a uma revisão manual das referências de todos os estudos incluídos, bem como revisões sistemáticas e metanálises anteriores para identificar quaisquer estudos relevantes adicionais do início até agosto de 2023.

Não houve restrição quanto ao idioma, data, tipo de documento, status de publicação, ou à geografia para a inclusão dos registros. A última busca foi conduzida em agosto de 2023. Os descritores foram identificados no Medical Subject Headings (MeSH), Descritores em Ciências da Saúde (DECS) e Embase Subject Headings (Emtree). A estratégia de busca foi adaptada com base nos descritores em cada banco de dados e está apresentada no material suplementar.

### Desfechos

O estudo avaliou os desfechos de eficácia, que envolveram a recorrência de FA. Além disso, o estudo analisou a recorrência específica de arritmias atriais, definida como a ocorrência composta envolvendo FA, *flutter* atrial ou taquicardia atrial, e avaliou o tempo médio dos procedimentos como parte da avaliação de eficácia.

Os desfechos de segurança de interesse foram relacionados às complicações maiores, as quais foram definidas como complicações pericárdicas, disfunção no nó sinusal, e fístula atrioesofágica.

### Critérios de eligibilidade

Foram estabelecidos os seguintes critérios de inclusão para a seleção dos estudos elegíveis: (1) ECRs; (2) estudos comparando ablação por cateter envolvendo IVP e IPP versus ablação por cateter com IVP somente; (3) pacientes que se submeteram ao procedimento de ablação para FA persistente; e (4) estudos com duração de seguimento de pelo menos 12 meses; (5) publicações relatando pelo menos um dos desfechos clínicos de interesse.

A escolha pelo tempo de acompanhamento de 12 meses foi determinada por uma revisão inicial da literatura relevante, realizada após um período de ‘blanking’ de três meses. Nossa análise excluiu estudos que caíssem nas seguintes categorias: (1) estudos que apresentassem métodos de alocação não randomizada; (2) estudos que não tivesse um grupo submetido a somente IVP; e (3) estudos envolvendo pacientes diagnosticados com FA paroxística.

### Seleção do estudo e extração de dados

Resultados da busca eletrônica de bancos de dados pré-definidos foram enviados ao Zotero. A seleção dos estudos e a extração dos dados foram realizadas independentemente por dois investigadores. Um terceiro revisor resolveu discordâncias. Para registros em duplicata, somente o mais recente foi incluído. Os autores inicialmente rastrearam títulos e resumos, e em seguida avaliaram textos completos para verificar se os estudos preenchiam os critérios de inclusão.

Foram extraídos dados sobre: informações do estudo (referência, país, local do estudo, número de participantes, amostra, período de acompanhamento, variáveis testadas, e desfechos principais), aspectos sociodemográficos (idade, sexo masculino), comorbidades (hipertensão, diabetes e insuficiência cardíaca), parâmetros clínicos adicionais (CHA_2_DS_2_-VASc score, fração de ejeção do ventrículo esquerdo, diâmetro atrial esquerdo) e métodos empregados para medir a associação estatística (risco relativo e diferença média).

Os termos de busca utilizados foram: “ *atrial fibrillation* ,” “ *pulmonary vein isolation* ,” “ *electrical posterior box isolation* ,” “ *posterior left atrial wall isolation* ,” “ *posterior wall isolation* ,” “ *left atrial posterior wall isolation* ,” e “electrical isolation of the left posterior wall”. A estratégia de busca completa encontra-se no Apêndice A suplementar.

### Avaliação da qualidade

A qualidade dos ECRs foi avaliada utilizando-se a ferramenta revisada Cochrane para risco de viés de ensaios randomizados (RoB2). A ferramenta emprega um sistema de escore que categoriza os estudos como tendo um risco alto, baixo ou incerto de viés em cinco domínios: viés de seleção, desempenho, detecção, atrito, e de relato.^18^

Dois avaliadores analisaram independentemente o risco de viés nos estudos selecionados. Possíveis fontes de viés em estudos randomizados incluem geração de sequência aleatória, sigilo na alocação, participantes e colaboradores cegos, avaliação dos desfechos às cegas, dados incompletos do desfecho, relato seletivo, entre outros. Três escores de sim, não, e incerto foram atribuídos a cada item mencionado, para se referir a um risco alto, baixo, e desconhecido, respectivamente. As avaliações da RoB 2 foram inseridas em uma planilha do Excel. Os revisores resolveram discrepâncias por discussão.

A certeza geral do corpo de evidência foi analisada usando a abordagem GRADE, levando em consideração o risco geral de viés, consistência dos efeitos, imprecisão, evidência indireta e viés de publicação. Em caso de preocupações sérias em qualquer um desses domínios, a qualidade da evidência foi diminuída na classificação. O julgamento geral da RoB2 foi incorporado na avaliação GRADE.

Para explorar o potencial de viés de publicação, construiu-se um gráfico de funil; essa análise incluiu a plotagem de estimativas de acordo com os pesos dos estudos.

### Metanálise

Os efeitos do tratamento foram expressos como razão de risco (RR), uma vez que todos os desfechos eram binários. As RRs agrupadas foram calculadas usando modelos de efeitos aleatórios com o estimador DerSimonian e Laird e o método de Mantel-Haenszel, já que se esperava uma heterogeneidade clínica. Diferenças médias foram usadas para analisar desfechos contínuos. A heterogeneidade nos efeitos dos estudos foi investigada usando o teste Q de Cochran e a estatística I2. Intervalos de predição não foram usados devido ao pequeno número de estudos em cada metanálise. A fim de se obter uma análise mais detalhada, foram realizadas subanálises pré-especificadas. Essas subanálises incluíram: (1) avaliação do tipo da ablação térmica empregada, e (2) uma análise focada em dados obtidos exclusivamente de estudos identificados como de risco de viés baixo ou incerto.

A significância da heterogeneidade foi determinada por valores p abaixo de 0,10 e valores de I^2^ acima de 25%. Em casos de heterogeneidade alta e baixa, foi usado um modelo de efeitos aleatórios de DerSimonian e Laird.

A análise estatística foi realizada usando o Review Manager 5.4.1. Um nível de significância de 5% foi adotado.

## Resultados

### Características do estudo e avaliação da qualidade

**Figura 1:**
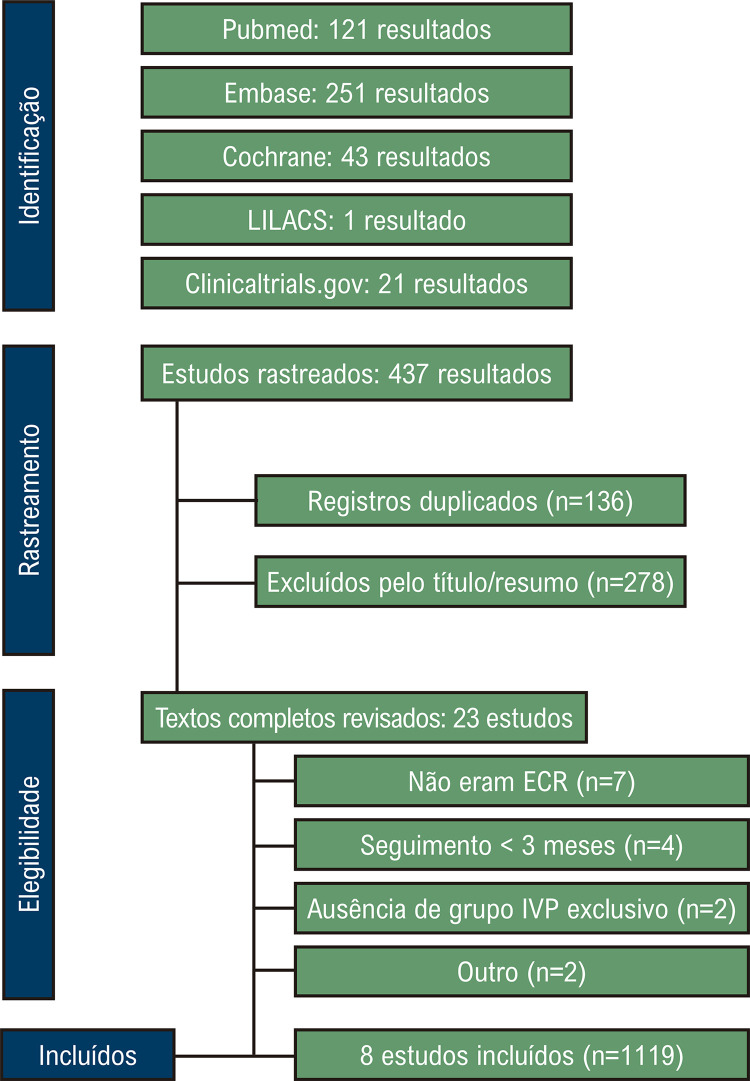
– Fluxograma PRISMA do rastreamento e da seleção dos estudos.

Como detalhado na Figura 1 , a fase inicial da identificação e de rastreamento gerou um total de 437 resultados. Após a aplicação dos critérios de elegibilidade, a análise primária identificou oito estudos envolvendo 1119 pacientes. Entre esses, 561 indivíduos (50,1%) foram submetidos a IPP adjuvante, e 558 indivíduos (49,9%) receberam ablação por cateter envolvendo IVP exclusivo. Todos os estudos incluídos foram randomizados quanto ao delineamento, com períodos de acompanhamento variando entre 12 e 22,5 meses. A idade média dos participantes variou entre 56 e 71 anos. As características basais dos estudos incluídos estão resumidas na Tabela 1 .

**Tabela 1:** – Características basais dos estudos incluídos

Estudo	Número de pacientes IPP+/IPP-	Seguimento^†^ (meses)	Homens (%) IPP +/ IPP -	Idade^†^ (anos) IPP +/ IPP -	HTN (%) IPP +/ IPP -	DM (%) IPP+/ IPP -	IC (%) IPP+/IPP-	CHA_2_DS_2_-VASc^†^ IPP+/IPP-	FEVE^†^ (%) IPP+/IPP-	DAE^†^ (mm) IPP+/IPP-
Ahn 2022^12^	50/50	15,3 ± 2	78/90	65/66	76/90	46/40	42/48	3/3	58/58	48/48
Aryana 2021^21^	55/55	12	64/60	68/71	64/67	25/27	24/29	2,4/2,8	60/61	44/44
Kim 2014^22^	60/60	12	77/68	56/58	42/48	13/15	15/23	ND	64/63	42/42
Kistler 2023^13^	170/168	12	77/76	66/66	50/44	10/10	26/31	2/2	56/55	46/44
Lee 2019^20^	102/105	16,2 ± 8,8	86/80	59/59	44/50	13/17	22/23	1,6/1,9	59/59	45/44
Pak 2020^‡24^	57/57	22,5±9,4	ND	ND	ND	ND	ND	ND	ND	ND
Wong 2023^‡14^	39/28	12,4 ± 3,0	79/71	68/68	69/79	15/29	28/36	2,5/2,9	51/53	48/46
Yamaji 2020^23^	24/33	ND	83/91	67/64	ND	ND	ND	1,8/1,5	60/60	42/46

^†^média ou mediana; ^‡^resumos de congresso; DM: diabetes mellitus; IC: insuficiência cardíaca; HTN: hipertensão; DAE: diâmetro atrial esquerdo; FEVE: fração de ejeção do ventrículo esquerdo; ND: não disponível; IPP +: isolamento da parede posterior adjuvante; IVP:isolamento exclusivo das veias pulmonares; todos os estudos adotaram um nível de significância de 5%.

Entre os estudos selecionados, seis utilizaram técnicas de ablação por radiofrequência,^13 , 14 , 20 , 22 - 24^ e dois usaram crioablação com balão.^12 , 21^ Sete estudos^12 - 14 , 20 - 23^ relataram medidas ecocardiográficas incluindo fração de ejeção ventricular esquerda e diâmetro atrial esquerdo no basal. Seis estudos^12 - 14 , 20 - 22^apresentaram informações sobre a porcentagem de pacientes com hipertensão e insuficiência cardíaca, além dos valores médios do escore CHA_2_DS_2_-VASc.

A avaliação da qualidade quanto aos principais desfechos está descrita na Tabela 2 . A qualidade global da evidência foi moderada.

**Tabela 2:** – Resumo dos resultados relacionados aos desfechos principais

	Número de pacientes	RR/DM (IC95%)	Certeza da evidência (GRADE*)
Recorrência de FA^12,13,20-22,24^	995	RR 0,66 (0,44, 0,98)	Moderada
Subgrupo: ablação por radiofrequência e recorrência de FA^13,20,22,24^	785	RR 0,81 (0,56, 1,19)	Moderada
Subgrupo: crioablação e recorrência de FA ^2,21^	210	RR 0,42 (0,20, 0,87)	Baixa
Recorrência de arritmia atrial^12-14,20-24^	1119	RR 0,83 (0,65, 1,06)	Moderada
Subgrupo: ablação por radiofrequência na recorrência de arritmia atrial^13,14,20,22-24^	909	RR 0,94 (0,73, 1,21)	Moderada
Subgrupo: crioablação por balão e recorrência de arritmia atrial^12,21^	210	RR 0,63 (0,44, 0,90)	Baixa
Complicações clínicas importantes^12-14,20,21,23,24^	999	RR 0,81 (0,42, 1,58)	Moderada
Média de tempo do procedimento^12-14,21,22,24^	1062	MD +23,37min (16,50, 30,23)	Moderada

Dados em número, RR ou DM (IC95%); FA: fibrilação atrial; DM: diferença média; RR: razão de risco; *GRADE: Grading of Recommendations Assessment, Development and Evaluation; certeza moderada: estamos moderadamente certos quanto à estimativa do efeito; o efeito verdadeiro é provavelmente próximo à estimativa do efeito, mas há a possibilidade de ser substancialmente diferente; baixa certeza: nossa certeza na estimativa do efeito é baixa; o efeito verdadeiro pode ser substancialmente diferente da estimativa do efeito.

### Análise agrupada de todos os estudos

**Figura 2:**
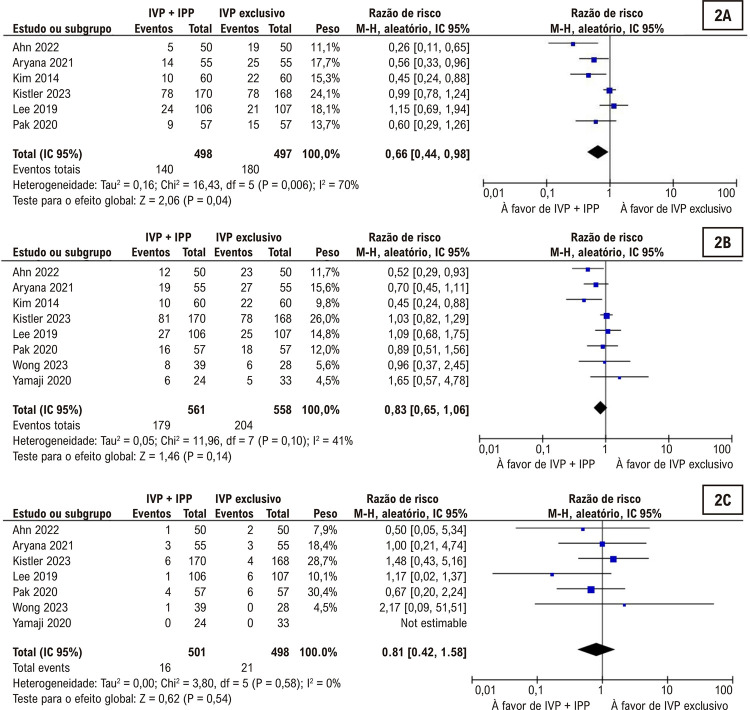
– A recorrência de fibrilação atrial foi mais baixa com isolamento da parede posterior (IPP) enquanto a recorrência de arritmia atrial (2B) e complicações clínicas importantes (2C) não foram significativamente diferentes entre os grupos; IVP: isolamento das veias pulmonares.

**Figura 3:**
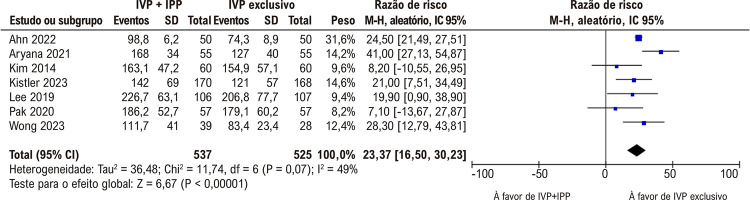
– As médias de tempos de procedimento foram mais baixas no isolamento exclusivo das veias pulmonares (IVP).

Entre os indivíduos que se submeteram ao IVP com IPP adjuvante, observou-se uma tendência à redução na recorrência de FA no grupo IVP + IPP ( Figura 2A ), sem diferença significativa nas complicações clínicas maiores ( Figura 2C ). No entanto, não foi observada diferença estatisticamente significativa entre os grupos quanto à recorrência de arritmia atrial ( Figura 2B ). Como esperando, os tempos médios de procedimento foram significativamente mais curtos no grupo submetido à ablação por cateter e somente a IVP ( Figura 3 ).

Para certificar a robustez desses achados, uma análise de sensibilidade foi realizada usando o método *leave-one-out* . Não foram observadas diferenças substanciais em nenhum dos desfechos considerados na análise agrupada.

### Subanálises nas populações selecionadas

**Figura 4:**
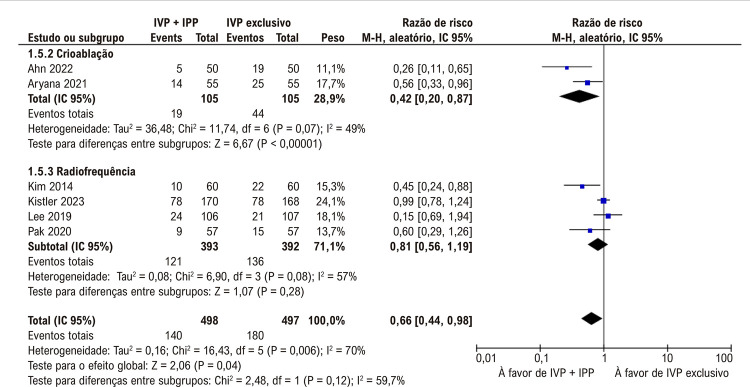
– Ablação por cateter usando isolamento das veias pulmonares (IVP) com isolamento da parede posterior (IPP) diminuiu a recorrência de fibrilação atrial com o uso de crioablação com balão, mas não com ablação por radiofrequência.

**Figura 5:**
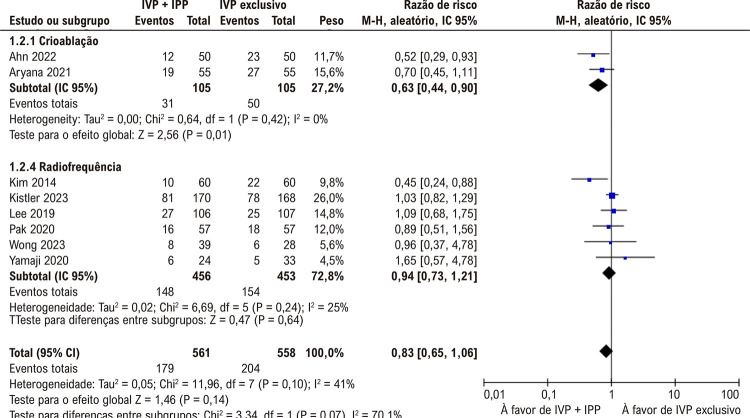
– Ablação por cateter usando isolamento das veias pulmonares (IVP) com isolamento da parede posterior (IPP) diminuiu recorrência arritmia atrial com o uso de crioablação com balão, mas não com ablação por radiofrequência.

Uma subanálise que segmentou os dados baseados no tipo do método de ablação utilizado, a crioablação em conjunto com IPP adjuvante demonstrou uma redução significativa na FA ( Figura 4 ). Contudo, não houve diferença significativa entre os grupos quando a ablação por radiofrequência foi empregada ( Figura 4 ). Quanto à recorrência de arritmia atrial, a crioablação com balão com IPP adjuvante levou à uma redução significativa, o que não foi observada com ablação por radiofrequência ( Figura 5 ).

**Figura 6:**
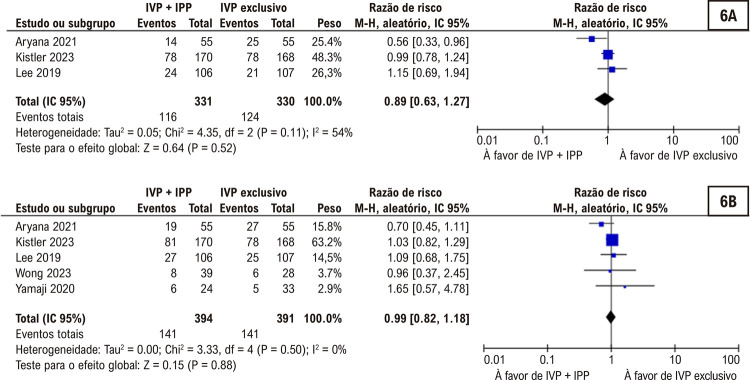
– Subanálise excluindo estudos com alto risco de viés não mostrou diferenças significativas entre os grupos em termos de recorrência de fibrilação atrial (6A) recorrência de arritmia atrial (6B).

Ao refinar a análise para incluir somente estudos classificados como de risco de viés baixo ou incerto, a subanálise revelou que não houve diferença entre os grupos em termos de recorrência de FA. O mesmo foi observado quanto à recorrência de arritmia atrial quando se considerou o IPP ( Figura 6B ).

### Avaliação da qualidade

A avaliação da qualidade de cada ECR encontra-se detalhada na Tabela S1 . Embora nenhum profissional que realizou a crioablação por cateter era cego durante os estudos, em cinco referências^13 , 14 , 20 , 21 , 23^ os avaliadores dos desfechos foram mantidos cegos quanto à alocação do tratamento do paciente. Em todos os três estudos classificados como de alto risco de viés,^12 , 22 , 24^ os avaliadores não eram cegos à randomização, induzindo, assim, a um risco potencial considerável de viés na interpretação do desfecho.

Como mostrado na Figura S1 , houve evidência prospectiva de viés de publicação, particularmente entre os estudos com menor RR. O gráfico de funil demonstra uma distribuição assimétrica de estudos de peso similar, principalmente no canto inferior esquerdo.

## Discussão

Nesta revisão sistemática e metanálise atualizada incluindo oito estudos e 1119 pacientes, comparamos principalmente a ablação por cateter com a ablação por cateter realizando tanto IVP como IPP. Os principais achados sugerem que o IPP do átrio esquerdo foi associado com menores taxas de recorrência de FA, mas maior média de tempo de procedimento. Entretanto, a inclusão do IPP pode não aumentar significativamente o período de tempo livre de ocorrência de arritmia atrial em pacientes com FA persistente, embora seja considerado um procedimento seguro em comparação ao IVP sozinho.

Vale ressaltar que uma análise mais detalhada do impacto do método de ablação, a crioablação por balão com IPP reduziu significativamente a recorrência tanto de FA como de arritmia atrial, enquanto não foi observado nenhum efeito significativo com a ablação por radiofrequência. Ao remover os estudos considerados de alto risco de viés, o IPP adjuvante não levou a uma diminuição na recorrência de nenhuma dessas duas condições. Consequentemente, a ablação com IPP poderia ser um procedimento adicional empregado nos pacientes com FA persistente. Resta provar se as novas técnicas de mapeamento sofisticadas que visam focos fora das veias pulmonares, juntamente com a ablação com IVP, podem melhorar os resultados de eficácia.

Enquanto o IPP adjuvante reduziu a recorrência de FA, a ocorrência de arritmias atriais continuou similar entre o grupo IVP + IPP e o grupo IVP. Essa observação pode ser devido à incidência aumentada de taquicardia atrial após isolamento completo da parede posterior do átrio esquerdo.^25 - 28^ A partir dessa perspectiva, as vantagens de diminuir a incidência de FA devem ser cuidadosamente avaliadas diante da possibilidade de os pacientes apresentarem outras arritmias atriais como a taquicardia atrial.^26 - 29^

A heterogeneidade e a inconsistência (I^2^ alto) foram observadas em certos desfechos de interesse e possivelmente poderiam ser atribuídas a vários fatores: (1) técnicas de ablação: a variabilidade nos tipos de técnicas de ablação utilizadas, isto é, crioablação ou radiofrequência. Métodos diferentes podem levar a desfechos variáveis dadas as diferenças no fornecimento de energia, características da lesão, e interações teciduais. (2) Variação do operador: diferentes operadores nos diferentes estabelecimentos de saúde, cada um com níveis variados de experiência e habilidades nos procedimentos de ablação por cateter, podem contribuir para heterogeneidade. (3) Método de monitoramento do ritmo: o uso de monitores de Holter intermitente versus monitoramento diário com dispositivo implantável pode introduzir heterogeneidade. Esses métodos distintivos de monitoramento do ritmo cardíaco podem influenciar a precisão da detecção de arritmia. (4) Risco de viés: a presença de um risco alto ou incerto de viés em alguns estudos podem contribuir para heterogeneidade. A variabilidade na qualidade do estudo e na metodologia pode impactar na confiabilidade dos resultados e na consistência dos desfechos.

A natureza complexa da FA persistente, caracterizada por degeneração progressiva e uma variedade de fenótipos, requer que sejam consideradas as características individuais dos pacientes e as estratégias de ablação, estendendo-se além da natureza da doença em si.^27 , 28^ Particularmente quando se considera um átrio com extenso remodelamento, a influência de outros vasos além da veia pulmonar sobre a manutenção da FA é bem conhecida. Nesse contexto, Verma et al.^2^ conduziram um ECR multicêntrico que explorou a eficácia da ablação extrapulmonar, e concluíram que a realização dessa técnica empiricamente não ofereceu benefícios. Diretrizes atuais^16 , 17 , 29^ também não recomendam ablação extrapulmonar empírica em pacientes com FA persistente. Assim, a ênfase passa para a necessidade de se identificar e executar a ablação extrapulmonar precisa para melhorar os desfechos clínicos em casos específicos. Ainda, as tecnologias de mapeamento existentes têm limitações na detecção de gatilhos. Espera-se que avanços futuros, tais como na técnica de crioablação^12 , 20 , 29 , 30^ ou na ablação por campo pulsado, melhore o tempo sem ocorrência de arritmia atrial em longo prazo. Além disso, uma abordagem personalizada, como ablação guiada por simulação, integrada com anatomia baseada em imagens, e a abordagem individualizada de áreas de baixa voltagem, poderia melhorar significativamente desfechos clínicos em pacientes com FA persistente.^30^

Metanálises prévias^10 , 11 , 15^ já haviam indicado a superioridade do IPP adjuvante em relação ao IVP exclusivo. Este estudo fornece evidência derivada de uma seleção de ECRs, reforçada por uma busca estratégica em múltiplas bases de dados. Ainda, a pesquisa conduziu uma avaliação da qualidade da evidência aplicando-se a metodologia FRAME. Esses resultados enfatizam a necessidade crítica de se aproveitar o potencial da ablação com IPP como uma opção terapêutica e destacam a importância de se adotar uma abordagem individualizada, focada no paciente. A permanência sem a ocorrência de arritmia atrial depende do conhecimento acerca do fenótipo atrial, da identificação dos gatilhos primários da FA, e da individualização do tratamento quanto a gatilhos extrapulmonares. Enquanto a busca de uma ablação mais extensa pareça atraente a fim de se melhorar os desfechos do procedimento, a ablação tecidual incompleta ou um bloqueio bidirecional inadequado pode levar a consequências pró-arritmogênicas, compostas por um alto risco de complicações associado com tempos mais longos de procedimento.

Diante desses resultados, ensaios futuros devem considerar sistematicamente a ablação além das veias pulmonares, utilizando o mapeamento atrial e uma abordagem fenotípica, com o auxílio da crioablação por balão ou ablação por campo pulsado. Essa abordagem poderia também categorizar diferentes subgrupos com base nas características fenotípicas e de remodelamento atrial comuns, direcionando, assim, pesquisas futuras. Dados clínicos e pré-clínicos apoiam nossos resultados, indicando que esses têm importância clínica e não passam de mera coincidência.^2 , 13 , 14 , 20 , 21 , 23^

É importante reconhecer as limitações deste estudo. Primeiro, a abordagem ao monitoramento do ritmo cardíaco após a ablação variou, sendo que monitores de Holter intermitentes foram utilizados em alguns estudos,^12 , 14 , 20 - 24^ e monitoramento diário e contínuo facilitado por dispositivos implantáveis em outros.^13^ Embora o monitoramento com dispositivos implantáveis seria o ideal, os custos associados seriam proibitivos. Ainda, nem sempre foi possível manter os avaliadores cegos quanto à randomização, incluindo alguns envolvidos na interpretação dos desfechos do estudo.

## Conclusão

Em pacientes diagnosticados com FA persistente, a inclusão de IPP demonstra benefícios potenciais para se atingir maior liberdade de ocorrência de FA em comparação a somente IVP. Nossos resultados enfatizam a necessidade de abordagens que consideram as características do paciente, a extensão do remodelamento atrial, e a utilização de técnicas efetivas de mapeamento, principalmente de locais extrapulmonares. Métodos abrangentes de IVP, tais como a crioablação por balão ou procedimentos por campo pulsado, podem revelar novas opções terapêuticas no manejo de pacientes com FA persistente.

## *Material suplementar

Para informação adicional, por favor,clique aqui


