# Supplementary material for: Eficácia e Segurança do Isolamento Adjuvante da Parede Posterior em Pacientes com Fibrilação Atrial Persistente: Uma Revisão Sistemática e Metanálise
Source: Arq Bras Cardiol. 2025 Jan 22;122(1):e20240472. [Article in Portuguese] doi: 10.36660/abc.20240472 (PMC11805571; doi:10.36660/abc.20240472)
Supplement: Supplementary file 1 [file 2024-0472_Supplementary_Material.pdf]

SUPPLEMENTARY MATERIAL

For

Efficacy and Safety of Adjunctive Posterior Wall Isolation in Patients with Persistent Atrial Fibrillation: A Systematic Review and Meta-Analysis

Novaes et al., 2023

TABLE OF CONTENTS

Appendix A. Search Strategy. 2

Table S1. Critical appraisal according to the Cochrane Collaboration's tool for assessing risk of bias in randomized trials for clinical outcomes. 2

Figure S1. Funnel plot for assessing publication bias 3

Appendix A. Search Strategy.

PubMed, Embase, Cochrane, LILACS and Clinicaltrials.gov:

"atrial fibrillation" AND "pulmonary vein isolation" AND ("electrical posterior box isolation" OR "posterior left atrial wall isolation" OR "posterior wall isolation" OR "left atrial posterior wall isolation" OR "electrical isolation of the left posterior wall")

Table S1. Critical appraisal according to the Cochrane Collaboration's tool for assessing risk of bias in randomized trials for clinical outcomes.

Study	Bias from randomization process	Bias due to deviations from intended interventions	Bias due to missing outcome data	Bias in the measurement of the outcomes	Bias in selection of the reported results	Overall risk of bias
Ahn 2022	Low	Some concerns	Low	High	Low	High
Aryana 2020	Low	Some concerns	Low	Low	Low	Some concerns
Kim 2014	Low	Low	Low	High	Some concerns	High
Kistler 2023	Low	Low	Low	Low	Low	Low
Lee 2019	Low	Low	Low	Low	Low	Low
Pak 2020	Low	Some concerns	Low	High	Some concerns	High
Wong 2023	Low	Low	Low	Some concerns	Some concerns	Some concerns
Yamaji 2020	Low	Some concerns	Low	Some concerns	Low	Some concerns

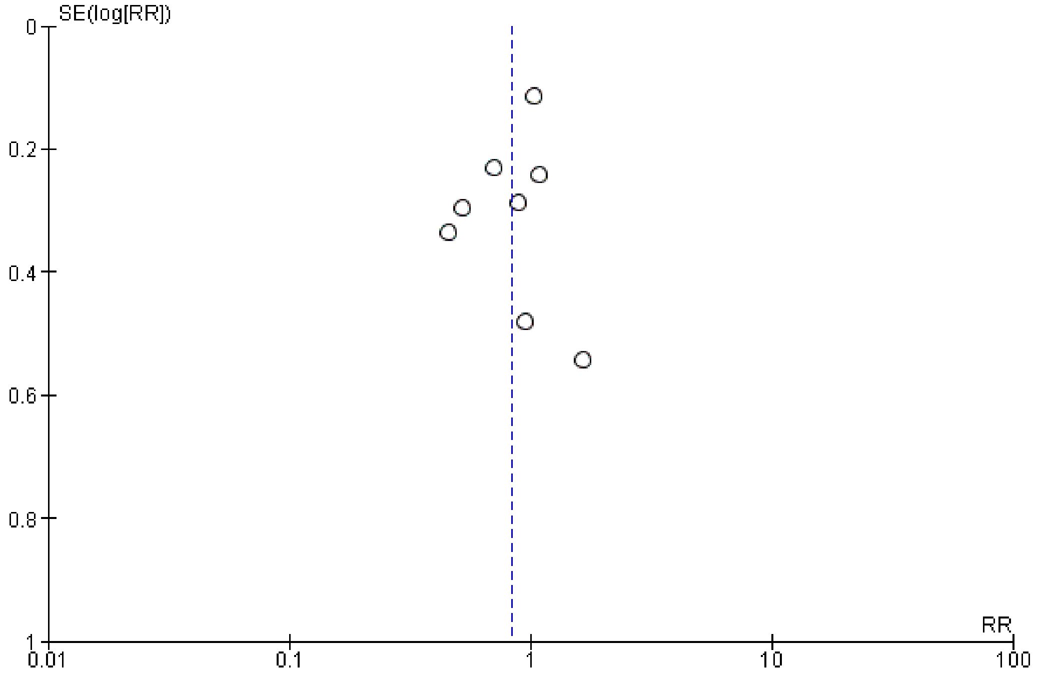

Figure S1. Funnel plot for assessing publication bias
