# Supplementary material for: Eficácia e Segurança do Isolamento Adjuvante da Parede Posterior em Pacientes com Fibrilação Atrial Persistente: Uma Revisão Sistemática e Metanálise
Source: Arq Bras Cardiol. 2025 Jan 22;122(1):e20240472. [Article in Portuguese] doi: 10.36660/abc.20240472 (PMC11805571; doi:10.36660/abc.20240472)
Supplement: Translation

# Translation: Efficacy and Safety of Adjunctive Posterior Wall Isolation in Patients with Persistent Atrial Fibrillation: A Systematic Review and Meta-Analysis

**DOI:** 10.36660/abc.20240472i

**Authors:** João Vitor Levindo Coelho Novaes, David de Pádua Brasil, Flavia Maria de Freitas Faria, Isadora Soares Bicalho Garcia, Camila Ribeiro Pimenta, Nathalia Sernizon Guimarães, Marcus Vinicius Bolivar Malachias

**Affiliations:** 1 Brazil Faculdade Ciências Médicas de Minas Gerais - Fundação Educacional Lucas Machado, Belo Horizonte, MG – Brazil; 2 Brazil Faculdade de Ciências da Saúde - Universidade Federal de Lavras, Lavras, MG – Brazil; 3 Brazil Departamento de Nutrição - Universidade Federal de Minas Gerais, Belo Horizonte, MG – Brazil

**Keywords:** Catheter Ablation, Atrial Fibrillation, Cardiac Electrophysiology

## Abstract

**Background:**

In patients with persistent atrial fibrillation (AF), addition of posterior wall isolation (PWI) to pulmonary vein isolation (PVI) is controversial.

**Objective:**

Compare PVI plus PWI versus PVI alone in patients with persistent AF.

**Methods:**

We searched PubMed (by MEDLINE), Embase, LILACS, CENTRAL (by Cochrane Library), and Clinicaltrials.gov databases for randomized trials comparing PVI + PWI and PVI alone in persistent AF. The outcomes were: (i) AF recurrence; (ii) composite of recurrent atrial arrhythmias (i.e., AF, atrial tachycardia, or atrial flutter); (iii) major clinical complications (i.e., pericardial effusion or tamponade, sinus node dysfunction, or atrioesophageal fistula); (iv) mean ablation time. Risk of bias and quality of evidence were evaluated using the Cochrane Risk of Bias 2.0 tool and GRADE, respectively. Statistical significance was set at 5%, and subgroup and sensitivity analyses were performed.

**Results:**

We included eight studies and 1119 patients, of which 561 underwent PVI + PWI. During follow-up (12 – 24 months), recurrence of AF was significantly reduced with adjunctive PWI (RR 0.66, 95% CI 0.44-0.98). Composite of recurrent atrial arrhythmias did not differ significantly (RR 0.83, 95% CI 0.65-1.06). Major clinical complications (RR 0.81, 95% CI 0.42-1.58) were similar, with PVI alone having a shorter mean procedure time (mean difference -23.37 minutes, 95% CI -30.23, -16.50).

**Conclusion:**

Adjunctive PWI appears to be effective in improving recurrent AF, but not recurrence of all atrial arrhythmias. Procedure time was longer with PVI + PWI without significant change in overall safety. Further studies should focus on long-term benefit.

## Introduction

The prevalence of AF is rising on the aging population, linked with metabolic syndrome. This increase in AF rates is associated with considerable mortality and morbidity risks, including heart failure and stroke.^1 - 3^Catheter ablation involving pulmonary vein isolation (PVI) tends to be less effective in patients with persistent atrial fibrillation (AF) compared to those with paroxysmal AF, as highlighted by previous studies.^4 , 5^

Early initiation of rhythm control is associated with slower AF progression and reduced cardiovascular and overall mortality risks compared to rate control. Notably, ablation techniques surpass drug therapy in maintaining sinus rhythm. However, in advanced AF stages, changes and remodeling in atrial substrate shift primary mechanisms away from the pulmonary veins to other structures within the left atrium.^6 , 7^

The posterior wall of the left atrium is believed to play a pivotal role in the pathophysiology of persistent AF, given the inclusion of the septopulmonary bundle and its embryological connection to the pulmonary veins.^8^Despite PVI procedures, reentrant circuits unrelated to the pulmonary veins may persist within the left atrium, thereby diminishing the efficacy of ablation procedure.^3 , 4 , 7 , 9^

In light of these considerations, posterior wall isolation (PWI) has gained a foothold as a technique to manage persistent AF. Following the publication of earlier meta-analyses^10 , 11^comparing adjunctive PWI to PVI in patients with persistent AF, subsequent randomized controlled trials (RCTs) have also been reported.^12 - 14^A recent meta-analysis^15^including randomized and non-randomized controlled trials, showed that PWI might significantly improve freedom from AF and overall atrial arrhythmia.

The 2020 European Society of Cardiology Guidelines acknowledged the potential of extensive ablation, including PWI, albeit its efficacy confirmation remains pending.^16^Similarly, the 2023 ACC/AHA Guidelines consider uncertain the assessment of endpoints beyond PVI.^17^Given the clinical relevance, we proposed to carry out an updated meta-analysis, exclusively considering RCTs and using the GRADE (Grading of Recommendations Assessment, Development and Evaluation) framework for evidence quality evaluation. The study aims to assess catheter ablation involving PVI with adjunctive PWI versus PVI alone in patients with persistent AF.

## Material and Methods

This systematic review and meta-analysis followed recommendations of the Cochrane Guidelines for Systematic Reviews of Interventions and was developed according to PRISMA.^18 , 19^The review protocol was registered at Open Science Framework (doi.org/10.17605/OSF.IO/AZ5GU).

### Search strategy

To identify clinical trials evaluating the effectiveness and safety of catheter ablation involving PVI with adjunctive PWI, as opposed to PVI alone in patients with persistent AF we searched four independent databases: PubMed (by MEDLINE), Embase, LILACS, Cochrane Central Register of Controlled Trials (CENTRAL) (by Cochrane Library). Additionally, we searched for registered clinical trials at the Clinicaltrials.gov and manually reviewed the references of all included studies, as well as prior systematic reviews and meta-analyses, to identify any additional relevant studies — from inception until August 2023.

There was no language, date, document type, publication status or geographic restriction for inclusion of records. The last search was conducted in August 2023. Descriptors were identified in Medical Subject Headings (MeSH), Descritores em Ciências da Saúde (DECS) and Embase Subject Headings (Emtree). The search strategy was adapted based on descriptors in each database and is presented in the Supplementary material.

### Outcomes

The study assessed efficacy outcomes, which encompassed the recurrence of AF. Additionally, the study analyzed the specific recurrence of atrial arrhythmias, defined as a composite occurrence involving AF, atrial flutter, or atrial tachycardia. and evaluated mean procedural times as part of the efficacy assessment.

The safety outcomes of interest revolved around major complications, which were defined as pericardial complications, sinus node dysfunction, or atrioesophageal fistula.

### Eligibility criteria

We established the following inclusion criteria for selection of eligible studies: (1) RCTs; (2) studies with comparison of catheter ablation involving PVI plus PWI *versus* catheter ablation with PVI alone; (3) patients who underwent ablation procedure for persistent AF; and (4) studies with a follow-up duration of at least 12 months; (5) publications reporting at least one of the clinical outcomes of interest.

The choice for a 12-month follow-up duration was determined by an initial review of relevant literature, conducted following a three-month blanking period. Our analysis excluded studies falling into the following categories: (1) those featuring non-randomized allocation methods; (2) those lacking a PVI alone group; and (3) those involving patients diagnosed with paroxysmal AF.

### Study selection and data extraction

Electronic search results from predefined databases were uploaded to Zotero. Study selection and data extraction was independently performed by two investigators. A third reviewer resolved any disagreements. For duplicate registrations, only the most recent one was included. Authors initially screened titles and abstracts, and subsequently assessed the full texts of the studies to determine whether they met inclusion criteria.

We extracted data on: study information (reference, country, study location, number of participants, sample, follow-up period, tested variables and main outcomes), sociodemographic aspects (age, male sex), comorbidities (hypertension, diabetes, and heart failure), additional clinical parameters (CHA_2_DS_2_-VASc score, left ventricular ejection fraction, left atrial diameter) and methods employed to measure statistical association (relative risk, and mean difference).

The search terms employed were: “atrial fibrillation,” “pulmonary vein isolation,” “electrical posterior box isolation,” “posterior left atrial wall isolation,” “posterior wall isolation,” “left atrial posterior wall isolation,” and “electrical isolation of the left posterior wall”. The complete search strategy can be found in Supplemental Appendix A.

### Quality assessment

The quality evaluation of RCTs was conducted using the revised Cochrane risk-of-bias tool for RCTs (RoB 2). The tool employs a scoring system that categorizes studies as having high, low, or unclear risk of bias across five domains: selection, performance, detection, attrition, and reporting biases.^18^

Two investigators independently assessed the risk of bias in the selected studies. Possible sources of bias in randomized trials include random sequence generation, allocation concealment, blinding of participants and personnel, blinding of outcome assessment, incomplete outcome data, selective reporting, among others. Three scores of yes, no, and unclear were given to each before mentioned item, referring to high risk, low risk, and unknown risk, respectively. We entered and organized our RoB 2 assessments on an Excel spreadsheet. Reviewers resolved discrepancies by discussion.

The overall certainty of the body of evidence was rated by using the GRADE approach, considering overall risk of bias, consistency of effect, imprecision, indirectness and publication bias. If there were serious concerns in any of these domains, we rated down the quality of evidence. We incorporated the overall RoB2 judgment into our GRADE assessment.

To explore the potential for publication bias, a funnel-plot was constructed; this analysis involved plotting point estimates in accordance with study weights.

### Meta-analysis

Treatment effects were expressed as risk ratios (RRs) as all outcomes were binary. Pooled RRs were calculated using random effect models with the DerSrlfi0monian and Laird estimator and the Mantel-Haenszel method, as clinical heterogeneity was expected. Mean differences were employed to analyze continuous outcomes. Statistical heterogeneity among studies’ effects was investigated by using Cochran Q test and I^2^statistic. Prediction intervals were not used due to the small number of studies in each meta-analysis. In order to provide a more detailed analysis, pre-specified subanalyses were carried out. These subanalyses included: (1) an examination of the type of thermal ablation employed, and (2) a focused analysis of data solely from studies identified as having low or uncertain risk of bias.

Significance for heterogeneity was determined by *p* -values below 0.10 and I^2^values surpassing 25%. In cases of both high and low heterogeneity, a DerSimonian and Laird random-effects model was employed.

The statistical analysis was conducted using Review Manager 5.4.1. A level of significance of 5% was adopted.

## Results

### Study characteristics and quality assessment

As detailed in Figure 1 , the initial phase of identification and screening yielded a total of 437 results. Following the application of eligibility criteria, the primary analysis identified eight studies involving 1119 patients. Among these, 561 individuals (50.1%) underwent adjunctive PWI, while 558 subjects (49.9%) received catheter ablation involving PVI alone. All the included studies were randomized in design, with follow-up periods ranging from 12 to 22.5 months. The mean age of participants ranged from 56 to 71 years. Baseline characteristics of included studies are summarized in Table 1 .

**Figure 1:** – PRISMA flow diagram of study screening and selection.

**Table 1:** – Baseline characteristics of included studies

Study	No. of Patients PWI+/PWI-	Follow-Up^†^(months)	Men (%) PWI+/PWI-	Age^†^(years) PWI+/PWI-	HTN (%) PWI+/PWI-	DM (%) PWI+/PWI-	HF (%) PWI+/PWI-	CHA_2_DS_2_-VASc^†^PWI+/PWI-	LVEF^†^(%) PWI+/PWI-	LAD^†^(mm) PWI+/PWI-
Ahn 2022^12^	50/50	15.3 ± 2	78/90	65/66	76/90	46/40	42/48	3/3	58/58	48/48
Aryana 2021^21^	55/55	12	64/60	68/71	64/67	25/27	24/29	2.4/2.8	60/61	44/44
Kim 2014^22^	60/60	12	77/68	56/58	42/48	13/15	15/23	NA	64/63	42/42
Kistler 2023^13^	170/168	12	77/76	66/66	50/44	10/10	26/31	2/2	56/55	46/44
Lee 2019^20^	102/105	16.2 ± 8.8	86/80	59/59	44/50	13/17	22/23	1.6/1.9	59/59	45/44
Pak 2020^‡24^	57/57	22.5±9.4	NA	NA	NA	NA	NA	NA	NA	NA
Wong 2023^‡14^	39/28	12.4 ± 3.0	79/71	68/68	69/79	15/29	28/36	2.5/2.9	51/53	48/46
Yamaji 2020^23^	24/33	NA	83/91	67/64	NA	NA	NA	1.8/1.5	60/60	42/46

^†^mean or median;^‡^conference abstracts; DM: diabetes mellitus; HF: heart failure; HTN: hypertension; LAD: left atrial diameter; LVEF: left ventricular eject fraction; NA: not available; PWI+: adjunctive posterior wall isolation; PWI-: pulmonary vein isolation alone. All studies adopted a level of significance of 5%.

Among the selected studies, six used radiofrequency ablation techniques^13 , 14 , 20 , 22 - 24^and two employed cryoballoon catheter ablation.^12 , 21^Seven studies^12 - 14 , 20 - 23^reported echocardiographic measurements including left ventricular ejection fraction and left atrium diameter at baseline. Six studies^12 - 14 , 20 - 22^provided information on the percentage of patients with hypertension, diabetes, and heart failure, along with the mean CHA_2_DS_2_-VASc score.

Quality assessed at the outcome level using GRADE methodology is presented in Table 2 . The overall quality of the evidence was moderate.

**Table 2:** – Summary of findings for the main outcomes

	Number of patients	RR/MD (95% CI)	Certainty of evidence (GRADE*)
AF recurrence^12,13,20-22,24^	995	RR 0.66 (0.44, 0.98)	Moderate
Subgroup: radiofrequency ablation in AF recurrence^13,20,22,24^	785	RR 0.81 (0.56, 1.19)	Moderate
Subgroup: cryoballoon ablation in AF recurrence^2,21^	210	RR 0.42 (0.20, 0.87)	Low
Atrial arrhythmia recurrence^12-14,20-24^	1119	RR 0.83 (0.65, 1.06)	Moderate
Subgroup: radiofrequency ablation in atrial arrhythmia recurrence^13,14,20,22-24^	909	RR 0.94 (0.73, 1.21)	Moderate
Subgroup: cryoballoon ablation in atrial arrhythmia recurrence^12,21^	210	RR 0.63 (0.44, 0.90)	Low
Major clinical complications^12-14,20,21,23,24^	999	RR 0.81 (0.42, 1.58)	Moderate
Mean procedure time^12-14,21,22,24^	1062	MD +23.37min (16.50, 30.23)	Moderate

Data are n, RR or MD (95% CI). AF: atrial fibrillation; MD: mean difference; RR: risk ratio. *GRADE: Grading of Recommendations Assessment, Development and Evaluation. Moderate certainty: we are moderately confident in the effect estimate; the true effect is likely to be close to the estimate of the effect, but there is a possibility that it is substantially different. Low certainty: our confidence in the effect estimate is low; the true effect might be substantially different from the estimate of the effect.

### Pooled analysis of all studies

Among individuals who underwent PVI with adjunctive PWI, there was a trend indicating reduction in AF recurrence in the PVI + PWI group ( Figure 2A ), without significant difference in major clinical complications ( Figure 2C ). However, no statistically significant difference was observed between groups concerning atrial arrhythmia recurrence ( Figure 2B ). As expected, mean procedural times were significantly shorter in the group undergoing catheter ablation with PVI alone ( Figure 3 ).

**Figure 2:** – Atrial fibrillation recurrence (2A) was lower with adjunctive posterior wall isolation (PWI), while atrial arrhythmia recurrence (2B), and major clinical complications (2C) were not significantly different between groups.

**Figure 3:** – Mean procedural times were lower when performing pulmonary vein isolation alone.

To ascertain the robustness of these findings, a sensitivity analysis was conducted using the leave-one-out method. Notably, no substantial differences emerged in the overall pooled analysis for any of the endpoints under consideration.

### Subanalyses in selected populations

A subanalysis that segmented data based on the type of ablation method utilized, cryoballoon ablation in conjunction with adjunctive PWI demonstrated a significant reduction in AF ( Figure 4 ). However, no significant difference between groups was found when radiofrequency ablation was employed ( Figure 4 ). Regarding atrial arrhythmia recurrence, cryoballon ablation with adjunctive PWI yielded a significant reduction, which was not observed with radiofrequency ablation ( Figure 5 ).

**Figure 4:** – Catheter ablation using pulmonary vein isolation with posterior wall isolation reduced atrial fibrillation recurrence when using cryoballoon, but not with radiofrequency ablation.

**Figure 5:** – Catheter ablation using pulmonary vein isolation with posterior wall isolation reduced atrial arrhythmia recurrence when using cryoballoon, but not with radiofrequency ablation.

Upon refining the analysis to include only studies evaluated as having low or uncertain risk of bias, the subanalysis revealed no substantial difference between groups in terms of AF recurrence ( Figure 6A ). Similarly, the impact on atrial arrhythmia recurrence remained statistically neutral when adjunctive PWI was considered ( Figure 6B ).

**Figure 6:** – Subanalysis excluding high risk of bias studies showed no significant difference between groups in terms of atrial fibrillation recurrence (6A) and atrial arrhythmia recurrence (6B).

### Quality assessment

Quality assessment of each RCT is detailed in Table S1 . Although no catheter ablation operators were blinded during the studies, in five references^13 , 14 , 20 , 21 , 23^outcome adjudicators were kept blinded to patient treatment allocation. In all three studies labeled as high overall risk of bias,^12 , 22 , 24^the operators were not blinded to randomization, thereby introducing a considerable potential risk of bias in outcome interpretation.

As depicted in Figure S1 , there has been prospective evidence of publication bias, particularly among lower-weighted studies associated with lower RRs. The funnel plot demonstrates an asymmetric distribution of similarly weighted studies, primarily in the lower left corner.

## Discussion

In this systematic review and updated meta-analysis including eight studies and 1,119 patients, we compared catheter ablation using PVI alone to catheter ablation performing PVI with PWI. Primary findings suggest that isolation of the posterior wall of the left atrium was associated with lower AF recurrence rates but longer mean procedural times. However, inclusion of PWI may not significantly increase freedom from atrial arrhythmia in patients with persistent AF, despite being considered a safe procedure when compared to PVI alone.

Notably, when scrutinizing the impact of the ablation method, cryoballoon ablation with PWI significantly reduced both AF and atrial arrhythmia recurrence, whereas no significant effect was observed with radiofrequency ablation. Remarkably, when removing studies assessed as having a high risk of bias, adjunctive PWI did not lead to a decrease in either AF or atrial arrhythmia recurrence. Consequently, PWI ablation could be an additional procedure employed in patients with persistent AF. Whether new sophisticated mapping techniques targeting non-pulmonary vein foci combined with PVI ablation can improve efficacy results remains to be proved.

While adjunctive PWI reduced the recurrence of AF, the overall occurrence of atrial arrhythmias remained similar between the PVI+PWI and PVI only groups. This observation could be due to an increased incidence of atrial tachycardia following the complete isolation of the posterior wall of the left atrium.^25 - 28^From this perspective, the advantages of decreasing AF incidence must be carefully balanced against the likelihood of patients experiencing other atrial arrhythmias, such as atrial tachycardia.^26 - 29^

Heterogeneity and inconsistency (high I^2^) were observed in certain endpoints of interest and could likely be attributed to several key factors: (1) ablation techniques: the variability in the types of ablation techniques used, *i.e.* cryoballoon or radiofrequency. Different methods may yield varying outcomes due to differences in energy delivery, injury characteristics, and tissue interactions. (2) Operator variation: different operators across various health care settings, each with varying levels of experience and skills in catheter ablation procedures, may contribute to heterogeneity. (3) Rhythm monitoring manner: the use of intermittent Holter monitors *versus* daily implantable device monitoring may introduce heterogeneity. These distinctive methods of rhythm monitoring may influence the precision of arrhythmia detection. (4) Risk of bias: the presence of high or uncertain risk of bias in some studies could contribute to heterogeneity. Variability in study quality and methodology can impact the reliability of results and the consistency of outcomes.

The complex nature of persistent AF, characterized by progressive degeneration and a variety of phenotypes, requires consideration of individual patient characteristics and ablation strategies, extending beyond the disease’s nature itself.^27 , 28^Particularly when confronting extensively remodeled atria, influence of extra-pulmonary vein triggers on AF maintenance is well-acknowledged. In this context, Verma et al.^2^conducted a multicenter RCT that explored the efficacy of extra-PV ablation, concluding that empirical extra-PV ablation did not offer benefits. Current guidelines^16 , 17 , 29^also do not recommend routine empirical extra-PV ablation in patients with persistent AF. Hence, the emphasis shifts towards the need to identify and precisely ablate extra-PV foci to enhance clinical outcomes in specific cases. Yet, the existing mapping technologies have limitations when it comes to trigger mapping. Future advancements, such as in cryoballoon^12 , 20 , 29 , 30^or pulsed field ablation, are expected to yield better long-term freedom from atrial arrhythmia outcomes. Moreover, a personalized approach, such as simulation-guided ablation integrated with image-based anatomy and individualized low-voltage area targeting, could significantly enhance clinical outcomes in persistent AF patients.^30^

Previous meta-analyses^10 , 11 , 15^had already indicated the superiority of adjunctive PWI over PVI alone. This study provides evidence derived from a selection of RCTs, reinforced by a search strategy across multiple databases. In addition, the research conducted a quality of evidence assessment with the GRADE framework. Altogether, the results emphasize the critical need to grasp the potential of PWI ablation as a therapeutic option and underscores the significance of adopting a patient-specific, targeted approach. Long-term freedom from atrial arrhythmia hinges on understanding the atrial phenotype, identifying primary AF triggers, and tailoring treatment for individual extra-PV triggers. While the pursuit of more extensive ablation might seem appealing to enhance procedural outcomes, incomplete tissue ablation or inadequate bidirectional block can yield proarrhythmogenic consequences, further compounded by high risk of complications associated with longer procedural times.

In light of our findings, we propose that forthcoming trials should systematically evaluate guided extra-PV ablation using atrial mapping and a phenotypical approach, with the aid of cryoballoon or pulsed-field ablation. This approach could also categorize different subgroups based on phenotypical and common atrial remodeling characteristics, thus guiding further research efforts. Both clinical and preclinical data offer support, indicating that our results are clinically meaningful and not a mere coincidence.^2 , 13 , 14 , 20 , 21 , 23^

It is important to acknowledge the limitations of this study. First, the approach to rhythm monitoring post-ablation varied, with intermittent Holter monitors used in some studies,^12 , 14 , 20 - 24^and daily or continuous monitoring facilitated by implantable devices in others.^13^While daily implantable device-based monitoring would be optimal, the associated costs can be prohibitive. Additionally, the blinding of operators to randomization was not always feasible, and in some instances, those interpreting study endpoints were also not blinded.

## Conclusion

In patients diagnosed with persistent AF, the inclusion of PWI demonstrates potential benefits for achieving greater freedom from AF when compared to the conventional PVI alone. Our findings emphasize the need for approaches that consider the patient’s characteristics, the extent of atrial remodeling, and the utilization of effective mapping techniques, especially to non-pulmonary vein sites. Comprehensive PVI ablation methods, such as cryoballoon or pulsed-field-based procedures, may unveil new therapeutic options to manage patients with persistent AF.

## *Supplemental Materials

For additional information, please click here.
